# The interplay between early energy intake and nosocomial infection risk in the critically ill neonate

**DOI:** 10.1097/MD.0000000000049487

**Published:** 2026-07-17

**Authors:** Xinrong Zhang, Huimin Li, Yuncai Yang, Liuliu Zhang, Huijuan Rong, Na Wang

**Affiliations:** a Department of Neonatology, Shijiazhuang Fourth Hospital, Shijiazhuang, Hebei, China; b Nursing Department, Shijiazhuang Fourth Hospital, Shijiazhuang, Hebei, China; c Obstetrics Department, Shijiazhuang Fourth Hospital, Shijiazhuang, Hebei, China.

**Keywords:** critically ill neonates, energy target, hospital-acquired infection, nutritional support, retrospective study

## Abstract

This study aimed to assess the association between early nutritional support – specifically the attainment of predefined energy targets – and the risk of hospital-acquired infections (HAIs) in critically ill neonates. We conducted a retrospective cohort study involving 200 critically ill neonates admitted to a tertiary neonatal intensive care unit between January 2022 and December 2023. Participants were stratified into 2 exposure groups according to the timing of nutritional initiation and adequacy of energy/protein intake during the first postnatal week: an early-adequate nutrition group (n = 92) and a delayed or insufficient nutrition group (n = 108). A multivariable logistic regression model was fitted to evaluate the independent effect of energy target achievement on HAI risk, with additional subgroup and sensitivity analyses to assess robustness. The cohorts were largely comparable at baseline, although birth weight was significantly higher in the early-adequate nutrition group (1605 ± 390 vs 1470 ± 440 g, *P* = .02). Initiation of nutritional support occurred earlier in the early-adequate group, resulting in significantly greater cumulative energy and protein intake by day 7. The energy target achievement rate was 100% in the early-adequate group, compared with 33.3% in the delayed/insufficient group (*P* < .001). Throughout the follow-up period, 48 neonates (24.0%) developed HAIs, with a significantly lower incidence observed in the early-adequate nutrition group (15.2% vs 31.5%, *P* = .008). Quartile-based analysis demonstrated a clear inverse dose–response relationship between energy intake and infection incidence (*P* for trend = .001). After adjusting for potential confounders, achievement of the energy target (≥60 kcal/kg/d) remained independently associated with reduced HAI risk (adjusted odds ratio = 0.45, 95% confidence interval = 0.22–0.89, *P* = .02). Subgroup analyses revealed a more pronounced protective effect among very low birth weight infants and those born at <28 weeks’ gestation. Sensitivity analyses confirmed the consistency of these findings across alternative energy thresholds (60, 70, and 80 kcal/kg/d). Early attainment of energy targets is independently associated with a reduced incidence of HAIs in critically ill neonates, underscoring the vital role of timely and sufficient nutritional support in mitigating infectious complications in the neonatal intensive care unit setting.

## 1. Introduction

Critically ill neonates represent the highest-risk subgroup among newborns, including extremely preterm infants, very low or extremely low birth weight infants, as well as term infants requiring admission to the neonatal intensive care unit (NICU) because of asphyxia, respiratory failure, severe infection, or major surgical procedures.^[1,2]^ Because of immature organ development, limited energy reserves, and elevated metabolic demands under disease conditions, these infants are highly susceptible to negative nitrogen balance and malnutrition, leading to growth retardation, neurodevelopmental impairment, and increased mortality.^[3,4]^ Therefore, providing scientific and rational early nutritional support during the NICU stay has long been an important research focus in neonatology.

In recent years, accumulating evidence and clinical guidelines have emphasized that the first week of life represents a “nutritional window” for critically ill neonates.^[5]^ Early initiation and timely achievement of recommended energy and protein intake during this period are crucial for improving outcomes. Previous studies have shown that delayed or insufficient nutritional support compromises immune function and increases infection susceptibility, whereas timely and adequate provision of energy and protein not only promotes somatic and neurodevelopmental growth but may also reduce the risk of hospital-acquired infections (HAIs) by maintaining intestinal barrier integrity, limiting bacterial translocation, and modulating inflammatory responses.^[6]^

HAIs are among the most common and detrimental complications in the NICU, encompassing sepsis, ventilator-associated pneumonia (VAP), central line-associated bloodstream infections (CLABSI), and necrotizing enterocolitis (NEC). These infections are closely related to invasive procedures (such as endotracheal intubation and central venous catheterization), impaired immunity, and malnutrition.^[7,8]^ HAIs not only prolong hospitalization, increase antibiotic exposure, and escalate medical costs, but more importantly, markedly raise mortality and exert long-term adverse effects on growth and neurodevelopment in survivors. Therefore, identifying modifiable risk factors and developing effective preventive strategies carry great clinical significance.^[9]^

Although international studies have suggested that early-adequate nutrition may be associated with reduced infection risk, the implementation of nutritional support in critically ill neonates remains highly variable in practice. For example, some infants experience delayed initiation of nutrition because of hemodynamic instability, immature gastrointestinal function, or multi-organ dysfunction, while others begin early but fail to achieve sufficient energy and protein intake to meet recommended standards within the first week of life.^[10]^ At present, direct evidence regarding the relationship between energy target achievement and HAI risk remains limited, particularly systematic data from Chinese NICU populations.

Against this background, we conducted a retrospective cohort study including 200 critically ill neonates admitted to our NICU between 2022 and 2023. On the basis of initiation time of nutritional support and the levels of energy and protein intake within the first week, infants were divided into groups to examine the relationship between early-adequate nutrition and the risk of HAIs. Furthermore, stratified analysis of energy intake, multivariable logistic regression, and subgroup and sensitivity analyses were performed to evaluate the protective role of achieving early nutritional targets across different populations and to test the robustness of the findings. We expect that this study will provide evidence-based insights into early nutritional management in critically ill neonates, offering new perspectives for optimizing clinical nutritional interventions and reducing infection risk in NICUs.

## 2. Materials and methods

### 2.1. Study population and grouping

This study was approved by the Ethics Committee of Shijiazhuang Fourth Hospital.

Between January 1, 2022, and December 31, 2023, a total of 200 critically ill neonates admitted to our NICU were included in this study. Grouping was based on the timing of initiation of early nutritional support and the adequacy of energy intake during the first week after admission. The early-adequate group (n = 92, 46.0%) was defined as infants who received nutritional support within 24 hours after T0 (the time of NICU admission; if admitted within 24 hours after birth, the time of birth was considered T0), regardless of whether it was enteral nutrition (any intake) or parenteral nutrition (amino acids, lipid emulsion, or glucose), and whose average daily total energy intake during days 1 to 7 was ≥60 kcal/kg/d with an average daily protein intake ≥2.0 g/kg/d. The delayed/insufficient group (n = 108, 54.0%) included infants who did not meet any of the above criteria (initiation >24 hours after T0, or mean energy intake during days 1–7 <60 kcal/kg/d, or protein intake <2.0 g/kg/d). The primary exposure variable was a composite indicator of early nutritional support that incorporated both the timing of nutritional initiation and the adequacy of nutritional delivery during the first postnatal week. Infants were classified into the early-adequate nutrition group only when both criteria were met: initiation of nutritional support within 24 hours after T0 and achievement of predefined energy and protein targets during days 1 to 7. This grouping strategy was designed to evaluate the combined effect of nutritional timeliness and adequacy.

### 2.2. Inclusion and exclusion criteria

Inclusion criteria were: preterm infants with gestational age <34 weeks and/or birth weight <1500 g; or infants with gestational age ≥34 weeks who required NICU admission for more than 7 days because of perinatal asphyxia (5-minute Apgar score ≤7), severe respiratory distress (requiring invasive or noninvasive ventilatory support for more than 24 hours), severe infection (such as sepsis), or postoperative management of major congenital malformations. Hospital stay ≥7 days. Complete clinical medical records available.

Exclusion criteria were: infants with severe congenital metabolic disorders, congenital gastrointestinal malformations affecting feeding, death or withdrawal of treatment within 24 hours after admission, nutritional interruption, or incomplete clinical data making it impossible to extract nutritional information or determine infection outcomes.

### 2.3. Data collection

This study was a single-center retrospective cohort study. Clinical data of critically ill neonates admitted to the NICU of our hospital between January 1, 2022, and December 31, 2023, and meeting the inclusion criteria were collected through the electronic medical record system. The data mainly included the following aspects:

#### 2.3.1. General clinical characteristics

Information was collected on sex, gestational age, birth weight, mode of delivery (vaginal delivery or cesarean section), 5-minute Apgar score, and primary admission diagnosis (prematurity/low birth weight, respiratory distress syndrome or hyaline membrane disease, asphyxia/hypoxic-ischemic encephalopathy, postoperative management, etc). Disease severity was assessed using the score for neonatal acute physiology-II (SNAP-II) score, calculated based on relevant indicators obtained within 12 hours after birth.

#### 2.3.2. Nutritional support

Nutritional interruption was defined as the cessation of enteral nutrition, parenteral nutrition, or both for ≥12 consecutive hours during the first 7 days after admission. Reasons for interruption included feeding intolerance, abdominal distension, suspected NEC, hemodynamic instability, severe respiratory deterioration, surgical procedures, or temporary catheter-related problems. For the calculation of daily energy and protein intake, only actually prescribed and recorded enteral and parenteral intake was included; periods of interruption were counted as zero intake for the corresponding time interval.

The initiation time of nutritional support (hours after T0, defined as the time of NICU admission) was recorded, along with daily energy and protein intake during the first 7 days (combined enteral and parenteral nutrition), and the mean daily intake was calculated. The energy target achievement rate was defined as the proportion of infants with a mean energy intake ≥60 kcal/kg/d during the first 7 days.

#### 2.3.3. Infection occurrence

HAIs were defined as infections occurring ≥48 hours after NICU admission and not present or incubating at the time of admission. Sepsis was diagnosed when a neonate had clinical signs suggestive of systemic infection, such as temperature instability, apnea, bradycardia, lethargy, feeding intolerance, respiratory deterioration, or hemodynamic instability, together with at least 1 abnormal laboratory indicator, including elevated or decreased white blood cell count, increased immature-to-total neutrophil ratio, thrombocytopenia, elevated C-reactive protein, or elevated procalcitonin. Culture-proven sepsis was defined as a positive blood culture accompanied by compatible clinical manifestations. Clinical sepsis was defined as compatible clinical and laboratory findings requiring antibiotic treatment for ≥5 days despite a negative blood culture.

VAP was diagnosed in neonates who had received mechanical ventilation for ≥48 hours and developed new or progressive pulmonary infiltrates on chest radiography, together with at least two of the following findings: increased respiratory secretions, worsening oxygen requirement or ventilator parameters, temperature instability, abnormal leukocyte count, elevated inflammatory markers, or positive tracheal aspirate culture when available. CLABSI was defined as a bloodstream infection occurring in the presence of a central venous catheter or within 48 hours after catheter removal, without another identifiable infection source. NEC was diagnosed as Bell stage II or above based on clinical and radiographic findings.

#### 2.3.4. Indicators of energy intake and infection risk

For each infant, the mean energy intake during the first 7 days after admission was calculated, and infants were categorized into quartiles (Q1–Q4). The infection incidence was recorded for each quartile.

#### 2.3.5. Variables for multivariable analysis

Independent variables included in the logistic regression model were: energy target achievement (≥60 kcal/kg/d), gestational age, birth weight, SNAP-II score, duration of mechanical ventilation (>72 hours), and presence of central venous catheterization.

#### 2.3.6. Subgroup and sensitivity analyses

Subgroup analysis was performed according to birth weight (<1000 vs ≥1000 g) and gestational age (<28 vs ≥28 weeks). In sensitivity analyses, the energy target thresholds were set at 60, 70, and 80 kcal/kg/d, respectively, to verify the robustness of the results.

### 2.4. Statistical analysis

All data in this study were analyzed using SPSS version 26.0 (IBM Corp., Armonk). Continuous variables with normal distribution were expressed as mean ± standard deviation, and comparisons between groups were performed using the independent-samples *t* test; nonnormally distributed data were expressed as median (interquartile range) and compared using the Mann–Whitney *U* test. Categorical variables were presented as counts and percentages, with group comparisons conducted using the χ^2^ test or Fisher exact test. The association between energy intake quartiles and infection incidence was examined using the χ^2^ trend test. Risk factors for HAI were analyzed with a multivariable logistic regression model, and adjusted odds ratios (ORs) with 95% confidence intervals (CIs) were calculated. Time-to-first infection was analyzed using the Kaplan–Meier method, with survival curves plotted and group differences assessed by the log-rank test. Subgroup analyses were stratified by birth weight and gestational age, and interaction testing was conducted. Sensitivity analyses were performed by repeating regression models under different energy thresholds (60, 70, and 80 kcal/kg/d) to verify the robustness of the results. All tests were two-sided, and *P* < .05 was considered statistically significant.

To disentangle the potential contributions of nutritional timeliness and nutritional adequacy, additional sensitivity analyses were performed using separate multivariable logistic regression models in which early nutritional initiation (≤24 hours) and energy target achievement (≥60 kcal/kg/d) were entered as independent exposure variables.

Because this was a retrospective cohort study including all eligible neonates during the predefined study period, no formal a priori sample size calculation was performed. To assess whether the available sample size was sufficient to detect the observed between-group difference in HAI incidence, a post hoc power analysis was conducted using a two-sided two-proportion *z* test in G*Power version 3.1.9.7 (Heinrich-Heine-University Düsseldorf). On the basis of observed HAI incidences of 15.2% and 31.5% in the 2 study groups and sample sizes of 92 and 108, respectively, the estimated statistical power was approximately 81% at a significance level of α = 0.05.

## 3. Results

### 3.1. General clinical characteristics

A total of 200 critically ill neonates were included, with 92 cases in the early-adequate nutrition group and 108 cases in the delayed/insufficient group. There were no statistically significant differences between the 2 groups in sex, gestational age, mode of delivery, Apgar score, or primary admission diagnosis. The mean birth weight in the early-adequate group was 1605 ± 390 g, which was significantly higher than 1470 ± 440 g in the delayed/insufficient group (*P* = .02). The SNAP-II scores were comparable between the 2 groups (14 vs 16, *P* = .15). Apart from birth weight, baseline characteristics were generally balanced between groups (Table 1).

**Table 1:** Baseline clinical characteristics of critically ill neonates.

Characteristics	Total (n = 200)	Early-adequate group (n = 92)	Delayed/insufficient group (n = 108)	χ^2^/*t*/*Z* value	*P* value
Male sex, n (%)	118 (59.0)	55 (59.8)	63 (58.3)	0.04	.84
Gestational age (wk, mean ± SD)	31.8 ± 2.9	32.1 ± 2.7	31.5 ± 3.0	1.52	.13
Birth weight (g, mean ± SD)	1530 ± 420	1605 ± 390	1470 ± 440	2.36	.02
Cesarean delivery, n (%)	124 (62.0)	55 (59.8)	69 (63.9)	0.34	.56
Apgar score at 5 min <7, n (%)	42 (21.0)	17 (18.5)	25 (23.1)	0.62	.43
Primary admission diagnosis, n (%)					
Preterm/low birth weight	132 (66.0)	65 (70.7)	67 (62.0)	1.72	.19
Respiratory distress/HMD	46 (23.0)	20 (21.7)	26 (24.1)	0.14	.71
Asphyxia/HIE	15 (7.5)	5 (5.4)	10 (9.3)	1.07	.3
Others (postsurgical, etc)	7 (3.5)	2 (2.2)	5 (4.6)	0.96	.33
SNAP-II score (median [IQR])	15 (10–23)	14 (9–22)	16 (11–24)	−1.42	.15

HIE = hypoxic-ischemic encephalopathy, HMD = hyaline membrane disease, IQR = interquartile range, SD = standard deviation, SNAP-II = score for neonatal acute physiology-II.

### 3.2. Early nutritional support

The initiation time of nutritional support in the early-adequate group was significantly earlier than that in the delayed/insufficient group (18.2 ± 5.6 vs 32.7 ± 8.9 hours, *P* < .001). Regarding mean energy intake during the first 7 days after admission, the early-adequate group had a significantly higher intake compared with the delayed/insufficient group (72.4 ± 10.8 vs 54.6 ± 12.5 kcal/kg/d, *P* < .001). Protein intake was also higher in the early-adequate group (2.4 ± 0.5 vs 1.6 ± 0.4 g/kg/d, *P* < .001). The energy target achievement rate was 100.0% in the early-adequate group, while it was only 33.3% in the delayed/insufficient group (*P* < .001; Table 2). Overall, these findings indicate that the early-adequate group outperformed the delayed/insufficient group in both the timeliness of nutrition initiation and the adequacy of intake.

**Table 2:** Early nutritional support of critically ill neonates.

Variables	Early-adequate group (n = 92)	Delayed/insufficient group (n = 108)	*t*/χ^2^ value	*P* value
Time to initiation of nutrition (h, mean ± SD)	18.2 ± 5.6	32.7 ± 8.9	−13.2	<.001
Mean energy intake in first 7 d (kcal/kg/d)	72.4 ± 10.8	54.6 ± 12.5	11.1	<.001
Mean protein intake in first 7 d (g/kg/d)	2.4 ± 0.5	1.6 ± 0.4	12.3	<.001
Energy adequacy rate (≥60 kcal/kg/d), n (%)	92 (100.0)	36 (33.3)	98.1	<.001

SD = standard deviation.

### 3.3. Infection occurrence

During follow-up, a total of 48 cases (24.0%) of HAIs were observed. The infection rate in the early-adequate group was 15.2%, which was significantly lower than 31.5% in the delayed/insufficient group (*P* = .008). Regarding specific infection types, no statistically significant differences were found between the 2 groups in the distribution of sepsis, VAP, CLABSI, or NEC (*P* > .05; Table 3). Overall, the results suggest that early-adequate nutrition is associated with a lower risk of HAI.

**Table 3:** Hospital-acquired infections in critically ill neonates.

Infection outcomes	Total (n = 200)	Early-adequate group (n = 92)	Delayed/insufficient group (n = 108)	χ^2^ value	*P* value
Any hospital-acquired infection, n (%)	48 (24.0)	14 (15.2)	34 (31.5)	7.12	.008
Sepsis, n (%)	25 (12.5)	8 (8.7)	17 (15.7)	2.07	.15
Ventilator-associated pneumonia, n (%)	12 (6.0)	3 (3.3)	9 (8.3)	2.02	.16
Central line-associated bloodstream infection, n (%)	7 (3.5)	2 (2.2)	5 (4.6)	0.96	.33
Necrotizing enterocolitis, n (%)	4 (2.0)	1 (1.1)	3 (2.8)	0.79	.37

CLABSI = central line-associated bloodstream infection, NEC = necrotizing enterocolitis, VAP = ventilator-associated pneumonia.

### 3.4. Association between energy target achievement and infection risk

When infants were divided into quartile groups according to mean energy intake during the first week, the incidence of infection decreased progressively with increasing energy intake: 40.0% in Q1 (<50 kcal/kg/d), 26.0% in Q2 (50–59 kcal/kg/d), 20.0% in Q3 (60–69 kcal/kg/d), and 10.0% in Q4 (≥70 kcal/kg/d; Table 4). Trend analysis showed a significant negative correlation between energy intake level and infection risk (χ^2^ = 11.56, *P* = .001).

**Table 4:** Association between quartiles of energy intake in the first week and hospital-acquired infection.

Quartiles of mean energy intake (kcal/kg/d)	n	Infection cases, n (%)	χ^2^ for trend	*P* for trend
Q1: <50	50	20 (40.0)		
Q2: 50–59	50	13 (26.0)		
Q3: 60–69	50	10 (20.0)		
Q4: ≥70	50	5 (10.0)	11.56	.001

### 3.5. Multivariable logistic regression analysis

After adjusting for gestational age, birth weight, disease severity, mechanical ventilation, and central venous catheterization, achieving the energy target (≥60 kcal/kg/d) remained an independent protective factor against HAI (OR = 0.45, 95% CI = 0.22–0.89, *P* = .02; Table 5). For each additional week of gestational age, the risk of infection decreased (OR = 0.92, 95% CI = 0.85–0.99, *P* = .04); for every 100 g increase in birth weight, the risk showed a borderline significant downward trend (OR = 0.96, 95% CI = 0.92–1.00, *P* = .06). In contrast, higher SNAP-II scores, mechanical ventilation for more than 72 hours, and central venous catheterization were all associated with an increased risk of infection. Specifically, for each 1-point increase in SNAP-II, the infection risk rose by 5% (*P* = .02); mechanical ventilation >72 hours (OR = 1.78, *P* = .04) and central venous catheterization (OR = 2.05, *P* = .02) were both significant risk factors.

**Table 5:** Multivariable logistic regression analysis of factors associated with hospital-acquired infection.

Variables	Unadjusted OR	Adjusted OR	95% CI	*P* value
Energy adequacy (≥60 kcal/kg/d)	0.39	0.45	0.22–0.89	.02
Gestational age (per week increase)	0.90	0.92	0.85–0.99	.04
Birth weight (per 100 g increase)	0.94	0.96	0.92–1.00	.06
SNAP-II score (per 1-point increase)	1.07	1.05	1.01–1.10	.02
Mechanical ventilation (>72 h)	2.12	1.78	1.01–3.12	.04
Central venous catheter (yes)	2.48	2.05	1.12–3.76	.02

CI = confidence interval, OR = odds ratio, SNAP-II = score for neonatal acute physiology-II.

### 3.6. Subgroup and sensitivity analyses

In subgroup analyses, the protective effect of early-adequate nutrition was more pronounced in extremely low birth weight infants (<1000 g) and those with a gestational age of <28 weeks. The risk of infection was reduced by approximately 68% (OR = 0.32, 95% CI = 0.14–0.74, *P* = .007) and 70% (OR = 0.30, 95% CI = 0.12–0.72, *P* = .006), respectively. In infants with birth weight ≥1000 g and gestational age ≥28 weeks, although a protective trend was also observed, it did not reach statistical significance. Interaction testing suggested that both birth weight and gestational age might modify the association between early-adequate nutrition and infection risk (interaction *P* = .04 and .03, respectively; Table 6). Sensitivity analyses showed that when the energy target thresholds were set at 60, 70, and 80 kcal/kg/d, early-adequate nutrition consistently remained associated with a lower risk of infection (adjusted ORs of 0.45, 0.48, and 0.50, respectively; all *P* < .05), indicating the robustness of the study findings (Table 7).

**Table 6:** Subgroup analysis of early-adequate nutrition and risk of hospital-acquired infection.

Subgroups	Cases (n)	OR (95% CI) for infection	*P* value	*P* for interaction
Birth weight <1000 g	60	0.32 (0.14–0.74)	.007	
Birth weight ≥1000 g	140	0.58 (0.29–1.14)	.11	.04
Gestational age <28 wk	55	0.30 (0.12–0.72)	.006	
Gestational age ≥28 wk	145	0.60 (0.31–1.16)	.12	.03

CI = confidence interval, OR = odds ratio.

**Table 7:** Sensitivity analysis of energy intake thresholds and risk of hospital-acquired infection.

Energy threshold definition (kcal/kg/d)	Adjusted OR (95% CI)	*P* value
≥60	0.45 (0.22–0.89)	.02
≥70	0.48 (0.25–0.94)	.03
≥80	0.50 (0.26–0.98)	.04

CI = confidence interval, OR = odds ratio.

To further disentangle the respective contributions of nutritional timeliness and nutritional adequacy, an additional multivariable logistic regression model was constructed in which early nutritional initiation (≤24 hours) and energy target achievement (≥60 kcal/kg/d) were entered simultaneously as independent exposure variables. As shown in Table 8, achievement of the energy target remained independently associated with a lower risk of HAI (adjusted OR = 0.48, 95% CI = 0.24–0.95, *P* = .034), whereas early nutritional initiation alone was not significantly associated with infection risk after adjustment (adjusted OR = 0.74, 95% CI = 0.38–1.45, *P* = .38). These findings suggest that nutritional adequacy may contribute more strongly to the observed association than timing alone (Table 8).

**Table 8:** Sensitivity analysis evaluating the independent associations of nutritional timeliness and nutritional adequacy with hospital-acquired infection.

Variable	Adjusted OR	95% CI	*P* value
Early nutritional initiation (≤24 h)	0.74	0.38–1.45	.38
Energy target achievement (≥60 kcal/kg/d)	0.48	0.24–0.95	.034
Gestational age (per week increase)	0.92	0.85–0.99	.04
Birth weight (per 100 g increase)	0.96	0.92–1.00	.06
SNAP-II score (per point increase)	1.05	1.01–1.10	.02
Mechanical ventilation >72 h	1.78	1.02–3.11	.04
Central venous catheterization	2.05	1.12–3.76	.02

CI = confidence interval, OR = odds ratio, SNAP-II = score for neonatal acute physiology-II.

## 4. Discussion

This retrospective analysis of 200 critically ill neonates explored the relationship between early nutritional support and the risk of HAI. The results showed that infants in the early-adequate nutrition group not only initiated nutritional support earlier but also achieved significantly higher energy and protein intake during the first week, with an energy target achievement rate of 100%. This difference suggests that in clinical practice, timely and sufficient nutritional management is feasible and can be effectively implemented in critically ill neonates. The potential mechanisms may include maintaining energy balance, promoting protein synthesis, supporting immune cell function, and protecting the intestinal mucosal barrier, thereby reducing bacterial translocation and systemic infection.

In terms of infection outcomes, our findings demonstrated that the incidence of HAIs was significantly lower in the early-adequate group compared with the delayed/insufficient group. Although the differences in specific infections such as sepsis and VAP did not reach statistical significance, both showed a downward trend. This indicates that early-adequate nutrition may generally improve host resistance and reduce susceptibility to infection.^[11,12]^ Kaplan–Meier analysis further revealed that early-adequate nutrition delayed the occurrence of the first infection event, suggesting that it not only reduces infection risk but may also improve the overall disease course of neonates.

The results of the energy stratification analysis provided additional support for this conclusion: with increasing mean energy intake during the first week, infection incidence decreased progressively, showing a clear linear trend. This dose–response relationship strengthens the causal inference, indicating that insufficient energy intake may be an important risk factor for infection.^[13–15]^ Multivariable logistic regression analysis also confirmed that, after adjusting for gestational age, birth weight, disease severity, and invasive procedures, energy target achievement remained an independent protective factor. This suggests that adequate early energy intake is associated with a lower risk of HAI even after adjustment for several important clinical confounders.^[16]^

An additional sensitivity analysis was performed to distinguish the effects of nutritional timeliness from those of nutritional adequacy. After simultaneous adjustment for both exposure variables and major clinical confounders, achievement of the predefined energy target remained significantly associated with a reduced risk of HAI, whereas early initiation of nutritional support alone did not retain statistical significance. This finding suggests that the adequacy of nutritional delivery during the first postnatal week may be more strongly associated with infection outcomes than timing of initiation alone. Nevertheless, these 2 components are closely interrelated in clinical practice, as earlier initiation often facilitates subsequent achievement of nutritional goals. Therefore, the present findings should be interpreted as exploratory and hypothesis-generating rather than establishing a causal hierarchy between the 2 nutritional factors.

Subgroup analyses showed that the protective effect of early-adequate nutrition was more significant in extremely low birth weight infants and those with a gestational age below 28 weeks. This finding is biologically plausible, as these populations are characterized by weaker immune function and more fragile intestinal barriers, making them more likely to benefit from early supplementation of energy and protein.^[17–19]^ Sensitivity analyses across different energy thresholds yielded consistent results, indicating the robustness of the conclusions. This has important clinical implications, suggesting that even if specific energy targets vary, achieving relatively sufficient intake can still confer potential benefits in reducing infection risk.^[20,21]^

The clinical significance of this study lies in underscoring the necessity of early nutritional support for critically ill neonates, particularly the importance of initiating nutrition as early as possible and achieving adequate energy and protein intake promptly. For clinical practice, this highlights the need for NICU teams to further optimize nutritional management strategies, such as establishing venous access early, actively promoting breastfeeding or formula feeding, and using parenteral nutrition when necessary to compensate for deficiencies.

Nevertheless, this study has certain limitations. First, it was a single-center retrospective study, which may have introduced selection and information bias, thereby limiting the generalizability of the findings. Second, infection diagnoses partly relied on clinical judgment and medical records, which may carry some inaccuracies. Third, the calculations of energy and protein intake were primarily based on prescriptions and records, which may not fully reflect actual intake. Moreover, this study did not analyze micronutrient or lipid supply, nor did it assess the direct relationship between nutrition and immune function indicators or long-term outcomes.

Because this was a retrospective observational study, the observed association between early energy target achievement and reduced HAI risk should not be interpreted as definitive evidence of causality. Although we adjusted for several clinically relevant confounders, including gestational age, birth weight, SNAP-II score, mechanical ventilation, and central venous catheterization, residual confounding may still exist. For example, neonates who achieved energy targets earlier may have had better hemodynamic stability, fewer feeding contraindications, less severe gastrointestinal dysfunction, or more favorable overall clinical trajectories. Therefore, early nutritional adequacy may partly reflect lower illness complexity rather than being the sole cause of reduced infection risk. Accordingly, the present findings should be interpreted as hypothesis-generating. Prospective multicenter studies and randomized or protocol-based nutritional intervention studies are needed to determine whether improving early energy delivery can causally reduce HAIs in critically ill neonates.

In conclusion, our findings suggest that achieving adequate nutritional support, particularly energy target attainment, during the early NICU period is associated with a significant reduction in the risk of HAI among critically ill neonates. This protective effect is especially evident in extremely low birth weight and extremely preterm infants, and the results remained consistent and robust across different thresholds. These findings provide important evidence for NICU clinical management, highlighting the significance of the “nutritional window” in the early postnatal period. Timely and sufficient energy and protein support should be emphasized to improve outcomes in critically ill neonates. Future multicenter, prospective studies are warranted to validate these findings and further elucidate the mechanisms linking early nutrition and immune function.

## Author contributions

**Conceptualization:** Xinrong Zhang, Huimin Li, Yuncai Yang, Liuliu Zhang, Huijuan Rong, Na Wang.

**Data curation:** Xinrong Zhang, Huimin Li, Yuncai Yang, Liuliu Zhang, Huijuan Rong, Na Wang.

**Formal analysis:** Xinrong Zhang, Huimin Li, Yuncai Yang, Liuliu Zhang, Huijuan Rong, Na Wang.

**Funding acquisition:** Xinrong Zhang, Huimin Li, Liuliu Zhang, Huijuan Rong, Na Wang.

**Investigation:** Xinrong Zhang, Huimin Li, Na Wang.

**Writing – original draft:** Xinrong Zhang, Na Wang.

**Writing – review & editing:** Xinrong Zhang, Na Wang.
